# The Mental Game of Tennis: A Scoping Review and the Introduction of the Resilience Racket Model

**DOI:** 10.3390/sports13050130

**Published:** 2025-04-23

**Authors:** Gerasimos N. Konstantinou, Stefan Kloiber, Daniel M. Blumberger

**Affiliations:** 1 Department of Psychiatry, University of Toronto, Toronto, ON M6J 1H4, Canada; stefan.kloiber@camh.ca (S.K.); daniel.blumberger@camh.ca (D.M.B.); 2 Centre for Addiction and Mental Health, Toronto, ON M6J 1H4, Canada; 3 Poul Hansen Centre for Depression, University Health Network, Toronto, ON M5T 2S8, Canada; 4 Temerty Centre for Therapeutic Brain Intervention, Toronto, ON M6J 1H4, Canada

**Keywords:** tennis, mental health, stress, resilience racket model

## Abstract

This review examines the relationship between tennis participation and mental health, highlighting both the psychological benefits and challenges associated with the sport. Using a retrospective, citation-based methodology, peer-reviewed studies published in English, French, and Greek between 2000 and March 2025 were included. The findings indicate that tennis participation is associated with reductions in depressive and anxiety symptoms, improved self-confidence, and enhanced resilience. However, competitive tennis also presents significant psychological demands, including elevated stress levels and susceptibility to maladaptive coping behaviors. To address these complexities, this review introduces the *Resilience Racket Model*, a conceptual framework representing the integration of physical readiness, psychological resilience, and systemic support. The model uses the metaphor of a tennis racket: the handle reflects foundational physical skills; the strings represent resilience components; the frame denotes environmental and organizational support; and the sweet spot signifies the optimal balance between physical and mental readiness. The review also highlights the effectiveness of psychological interventions, such as cognitive-behavioral therapy and mindfulness, in supporting athlete well-being. These findings advocate for a holistic approach to athlete development, emphasizing parity between mental health and physical training, and call for further research into tailored, sport-specific mental health interventions in tennis.

## 1. Introduction

Physical activity, exercise, and sports play distinct roles in human movement and physical exertion, serving various purposes and involving different levels of structure and competition [1]. Physical activity encompasses not only structured movements but also general activities integrated into daily life that involve bodily movements produced by skeletal muscles [2]. It is vital in diminishing the risks of a spectrum of physical health issues, including obesity, cardiovascular diseases, and metabolic dysfunctions [3]. Additionally, different types of physical activity contribute to mental health well-being, manifesting in heightened levels of happiness and tranquility alongside a reduction in distress, cumulatively fostering an improved quality of life [4,5,6].

Exercise, a subset of physical activity, is characterized by planned, structured, and repetitive bodily movement to improve or maintain one or more components of physical fitness [2]. Sports, on the other hand, involve physical activity that is usually competitive, governed by a set of rules or customs, and often engaged in socially [7]. Within the realm of sports, a further distinction is made between individual competitive sports and team sports, each with unique characteristics that influence the nature of the activities involved [8,9].

The extant literature has examined the symbiotic relationship between sports participation and mental well-being, aiming to delineate the complex mechanisms by which physical involvement in sports influences mental health [10]. This is particularly relevant for disabled athletes, where psychological readiness and concentration—both linked to mental processes—play a central role in optimizing performance [11]. Martin-Rodriguez et al., through a detailed analysis of neurobiological underpinnings and therapeutic applications, delve into the significant impact of sports on mental health, highlighting its role in emotion regulation, resilience enhancement, and cognitive function improvement [12]. A meta-analysis of 11 prospective studies involving over 69,000 participants found that higher physical activity levels significantly reduced the risk of developing depression by 26%, anxiety by 26%, and psychotic disorders by 27% [13].

Tennis, recognized as a sport that demands high physical and cognitive engagement, is an exemplary case study in the intersection of athletic performance and mental health within competitive contexts [14]. “The Inner Game of Tennis” by Timothy Gallwey is a seminal work that delves into the psychological aspects of sports performance, particularly focusing on tennis [15]. Gallwey introduces the concept of the “inner game,” which refers to the mental and emotional aspects of sports performance that often go unnoticed or unaddressed. He argues that mastering the inner game—cultivating focus, resilience, and mental clarity—is just as crucial as developing physical skills in sports. From a biomechanical perspective, tennis necessitates a comprehensive range of motor skills, including high-velocity serves, precise volleying, and dynamic lateral movements [16]. These actions require acute neuromuscular coordination, cardiovascular endurance, and muscle strength, highlighting the sport’s physical complexity. Additionally, tennis demands significant psychological resilience and strategic thinking. Athletes must maintain concentration and emotional control amidst the pressures of competition, demonstrating a capacity for acute stress management [17,18,19].

Despite the special characteristics, the numerous benefits, as well as the unique stressors, and the demands of the tennis players’ lifestyle, there is a paucity of research investigating the mental health challenges faced by this population. This review aims to critically examine the existing literature and contribute to the current knowledge base by offering insights for optimizing the mental health benefits of this popular sport. By synthesizing the findings from relevant studies, we formulate evidence-based recommendations for tennis federations, coaches, and players, emphasizing promoting and supporting mental health and well-being within the sport. This review adopts a comprehensive approach to explore the relationship between tennis and mental health, focusing on the psychological demands, mental health support strategies, and the effectiveness of psychological interventions in bolstering athlete well-being. It underscores the necessity for targeted mental health interventions and comprehensive support structures within sports organizations and broader societal frameworks to counteract mental health challenges faced by tennis athletes. Finally, this review introduces the *Resilience Racket Model*, a novel conceptual framework integrating physical, psychological, and environmental elements conceptualizing the synergy of physical skills and mental resilience in tennis athletes.

## 2. Materials and Methods

This study employed a scoping review methodology following the Preferred Reporting Items for Systematic Reviews and Meta-Analyses extension for Scoping Reviews (PRISMA-ScR) guidelines to systematically map the available evidence on mental health in tennis [20]. This approach was chosen to address the broad nature of the topic and to identify key concepts, gaps in research, and types of evidence available.

A comprehensive search was conducted in PubMed, Web of Science, and Scopus databases for peer-reviewed articles published in English between January 2000 and March 2025. The search strategy utilized a combination of terms related to tennis, mental health, and psychological factors. Specifically, the search algorithm included “tennis” AND (“mental health” OR “psychological health” OR “well-being” OR “depression” OR “anxiety” OR “stress” OR “burnout” OR “resilience” OR “mental toughness” OR “psychological skills” OR “cognitive performance”).

**Figure 1:**
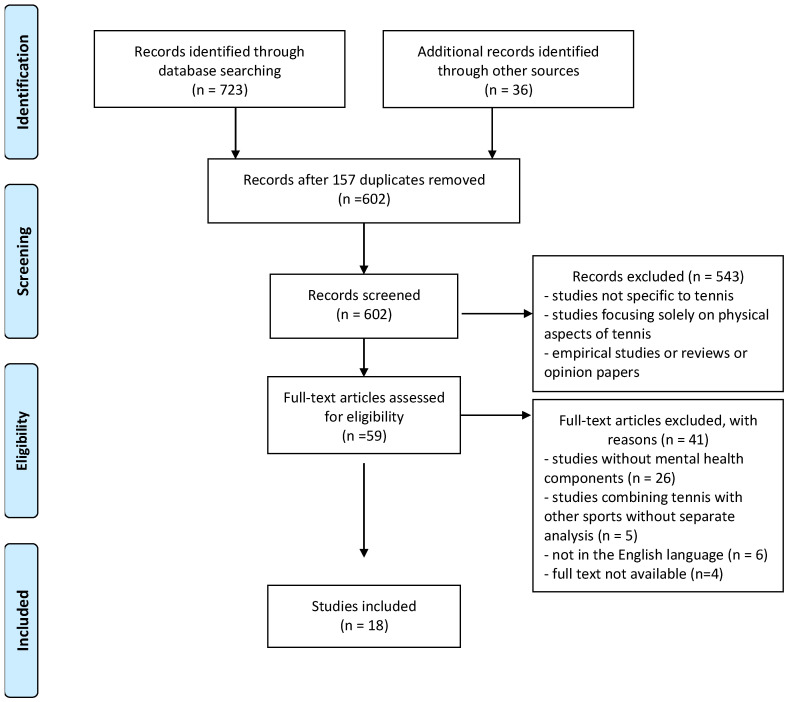
PRISMA flowchart of the selection of studies.

The inclusion and exclusion criteria are summarized in Table 1. Two independent reviewers screened titles and abstracts, followed by a full-text review of potentially eligible articles. Discrepancies were resolved through discussion with a third reviewer (Figure 1). Data extraction was performed using a standardized form, capturing study characteristics, population details, mental health outcomes, psychological interventions, and key findings.

Given the heterogeneity of the included studies, a narrative synthesis approach was adopted to summarize and interpret the findings. The synthesis was organized into thematic areas aligned with our research questions, focusing on the psychological demands of tennis, the prevalence of mental health issues among tennis players, and the effectiveness of psychological interventions in this population.

Based on the synthesized evidence, we developed the *Resilience Racket Model* as a conceptual framework to integrate the physical, psychological, and environmental factors influencing mental resilience in tennis. The model was iteratively refined through discussions among members of the research team.

## 3. Results

The present review identified 16 peer-reviewed studies published between January 2000 and March 2025 that examined the relationship between tennis and mental health outcomes. These studies employed diverse research designs, including randomized controlled interventions, quasi-experimental protocols, and large-scale observational analyses, thereby offering a robust and multi-dimensional view of psychological functioning in tennis populations. Study cohorts ranged from adolescent recreational players to elite professional athletes, allowing for the examination of developmental and high-performance contexts (Table 2).

Consistent patterns emerged regarding the mental health benefits of tennis engagement. Yazici et al. demonstrated that structured tennis training was associated with significant reductions in depressive and anxiety symptoms in a non-clinical university population, indicating broad-spectrum improvements in emotional and cognitive functioning [21]. These improvements were accompanied by significant reductions in sub-dimensions of psychological distress, such as interpersonal sensitivity, phobic anxiety, and paranoid ideation, suggesting a broad-spectrum impact of tennis participation on emotional and cognitive functioning.

Longitudinal associations between tennis engagement and psychological adaptation were further supported by Sun et al., who found that perceived social support played a mediating role in the relationship between tennis exposure, depressive symptoms, and prosocial behavior [22]. The psychological attributes necessary for competitive tennis success were explored in depth by Cowden et al. (2016), who demonstrated that mental toughness was positively associated with resilience and inversely related to perceived stress [23]. Regression analyses further confirmed that resilience sub-components were significant predictors of mental toughness, underscoring the dynamic interplay between dispositional resilience and the capacity for sustained performance under stress. Complementary evidence by Cowden et al. (2014) reinforced this framework, revealing that learned resourcefulness served as a key determinant of self-assessed mental toughness [24]. The authors suggest that the absence of a significant relationship between competitive trait anxiety and mental toughness in that study further indicates that psychological skills training targeting adaptive coping strategies may be more efficacious than interventions solely aimed at anxiety reduction.

At the elite level, psychological vulnerabilities associated with the demands of professional tennis were evidenced in the work of Marazziti et al., who identified elevated obsessive–compulsive tendencies among actively competing athletes compared to non-athlete controls [25]. As per the authors, this finding is indicative of the cognitive rigidity and compulsive preoccupations that may accompany high-performance environments characterized by perfectionism and continuous scrutiny. Notably, depressive symptoms did not differ between groups.

Performance dynamics under situational pressure were rigorously quantified in the large-scale analysis by Harris et al., which examined over 650,000 points from Grand Slam tournaments [26]. Their findings demonstrated a cumulative effect of psychological pressure, with prior unforced errors increasing the likelihood of subsequent errors, thereby highlighting the critical role of attentional control and emotional regulation in mitigating performance degradation during high-stakes moments. Lewis et al. conducted a qualitative case study examining the emotional experiences of adolescent tennis players during competitive tournaments [27]. The study identified a complex range of positively and negatively valenced emotions influenced by perceived performance, opponent behavior, match outcomes, and situational criticality. Participants reported using individualized coping strategies, including breathing techniques, cognitive reframing, and pre-performance routines. Notably, the study found that both positive and negative emotions could facilitate performance when appraised constructively.

Psychological interventions targeting tennis performance and mental health have shown promising effects [28]. For instance, evidence indicates that mindfulness-based protocols enhance concentration and focus, although the research is still scarce [29,30]. Morais et al. found that structured psychological skills training programs targeting anxiety regulation and self-confidence led to measurable improvements in these constructs, accompanied by better competitive outcomes [31]. The role of self-talk as a cognitive strategy was explored by Hatzigeorgiadis et al., whose controlled intervention study demonstrated that motivational self-talk significantly increased self-confidence and decreased cognitive anxiety, with improvements translating into enhanced task performance [32].

Psychological profiles in developmental populations were examined by Mourtzios et al., who observed gender-specific patterns, with girls exhibiting higher motivational and commitment scores and boys demonstrating superior concentration during match play [33]. Based on these results, the authors suggested that psychological training for youth athletes may benefit from gender-sensitive approaches tailored to distinct psychological strengths and vulnerabilities.

Finally, Taylor et al. (2008) contributed foundational insights into the psychological strategies employed by U.S. Olympic tennis players, revealing the systematic use of mental preparation techniques such as goal setting, imagery, and refocusing strategies as standard practice among elite performers [34]. Puente-Díaz (2013) expanded on the psychological underpinnings of performance by examining the complex interrelations between goal orientations, fear of failure, and self-confidence in competitive tennis players, further underscoring the multifaceted nature of mental health determinants in this sport [35]. The relationships between fear of failure, perfectionism (personal standards and concern over mistakes), achievement goals, and affective outcomes in 204 competitive tennis players aged 12–17 were examined. Using structural equation modeling, the study found that personal standards positively predicted mastery-approach and performance-approach goals, while concern over mistakes was positively associated with performance-avoidance goals. Fear of failure showed a significant negative relationship with mastery-approach goals. Mastery-approach goals significantly predicted enjoyment and hope, while performance-approach goals positively influenced hope. The study concludes that cultivating mastery-approach goals, supported by high personal standards rather than concern over mistakes, fosters positive achievement emotions essential for sustained motivation and performance in competitive tennis settings.

**Table 2:** Studies focused on tennis and mental health.

Study	Sample	Results	Conclusion
Mamassis & Doganis (2004) [28]	9 elite junior tennis players	Self-confidence increased by 7%; anxiety scores were significantly reduced (*p* < 0.05).	Structured mental training reduces anxiety and improves psychological readiness in junior tennis athletes.
Puente-Díaz (2013) [35]	204 competitive tennis players (ages 12–17)	Personal standards positively predicted mastery-approach goals (γ = 0.34, *p* < 0.05); fear of failure was negatively associated with mastery-approach goals (γ = −0.49, *p* < 0.05). Mastery-approach goals predicted enjoyment (β = 0.42, *p* < 0.05) and hope (β = 0.52, *p* < 0.05).	Emphasizing mastery-approach goals and high personal standards, rather than fear of mistakes, fosters positive achievement emotions, motivation, and sustained psychological well-being in competitive tennis.
Cowden et al. (2014) [24]	16 elite collegiate tennis players	Learned resourcefulness correlated strongly with mental toughness (r = 0.63; *p* < 0.01).	Resourcefulness enhances mental toughness and emotional regulation in competitive tennis players.
Casagrande et al. (2018) [36]	130 elite junior tennis players	Category 18yo had the highest burnout (U = 252.5, *p* = 0.001), linked to emotional exhaustion and reduced accomplishment.	Burnout risk increases with age and professional transition; proactive psychological monitoring is essential.
Yazici et al. (2016) [21]	76 university students	Depression was reduced by 24%; anxiety was reduced by 18% after tennis training.	Tennis effectively reduces depression and anxiety, supporting mental well-being in young adults.
Hatzigeorgiadis et al. (2009) [32]	72 young competitive tennis players	Self-confidence rose from 1.59 to 1.89 (*p* = 0.002); cognitive anxiety decreased from 1.25 to 0.89 (*p* = 0.031).	Motivational self-talk significantly reduces cognitive anxiety and improves self-confidence in youth tennis.
Cowden et al. (2016) [23]	351 competitive tennis players	Mental toughness correlated with resilience (r = 0.59; *p* < 0.001) and inversely with stress (r = −0.44; *p* < 0.001).	Psychological resilience and mental toughness act as protective factors for tennis players under competitive stress.
Lewis et al. (2017) [27]	4 adolescent competitive tennis players (aged 12–15)	Observed emotional fluctuations in competition, with both positive and negative emotions influencing performance depending on appraisal and coping strategies used.	Emotional responses in youth tennis are dynamic and context-dependent; individualized coping strategies and emotional regulation training are essential for resilience and competitive success.
Hoja & Jansen (2019) [30]	16 amateur tennis players	Concentration difficulties decreased by 12% (*p* < 0.05); perceived stress was reduced.	Mindfulness training reduces anxiety and improves focus during tennis performance.
Morais & Gomes (2019) [31]	11–14-year-old tennis athletes	Mental toughness increased by 9.6% after structured pre-service routines.	Pre-performance routines enhance emotional control and confidence in youth tennis players.
Peraita-Costa et al. (2020) [37]	94 junior tennis players (8–15 years)	Burnout more prevalent in girls (38%) vs. boys (29%), associated with mental exhaustion.	Gender differences in burnout risk require tailored psychological interventions for young tennis athletes.
Marazziti et al. (2021) [25]	25 current/former professional tennis players	Active players showed higher obsessive–compulsive scores (OCI-R = 19.8 ± 6.3 vs. 13.2 ± 4.9 in controls).	Professional tennis fosters compulsive tendencies under stress; psychological care should be part of athlete management.
Harris et al. (2021) [26]	Elite-level tennis matches (N = N/A)	Unforced error rates increased by 15% after prior mistakes, linked to pressure-induced attentional lapses.	Psychological pressure triggers error spirals in tennis; attentional and stress-control training are essential.
Marazziti et al. (2023) [38]	25 current/former professional tennis players	24% of active players reported problematic gambling behaviors related to anxiety and isolation.	Professional tennis players may develop behavioral addictions; structured psychological support is required.
Filipas et al. (2024) [39]	10 male tennis players (mean age 18 ± 4)	Second-serve accuracy errors increased by 13.2% under mental fatigue.	Cognitive fatigue impairs precision and confidence; mental load management is crucial before tennis matches.
Mourtzios et al. (2024) [33]	40 young tennis players (11–14 years)	Boys scored higher in concentration (M = 3.9); girls scored higher in emotional control (M = 4.1).	Gender-specific psychological training optimizes mental development and performance readiness in youth tennis.
Güler & Abdioglu (2025) [40]	206 recreational tennis players (during pandemic)	Stable SF-12 mental health scores (mean = 51.5 ± 6.2) despite pandemic conditions.	Recreational tennis supports emotional stability and mental health resilience during times of crisis.
Sun et al. (2025) [22]	150 general college students who play tennis and 150 professional tennis players	Tennis participation inversely related to depression (β = −0.234, *p* < 0.001); stronger prosocial behaviors observed.	Tennis enhances psychological well-being and social support in university populations, reducing depression symptoms.

## 4. Discussion

This review of studies spanning over the last two decades elucidates the complex and bidirectional relationship between tennis participation and mental health. The findings consistently demonstrate that tennis contributes to psychological well-being across both recreational and competitive contexts. Simultaneously, participation in competitive and elite tennis imposes substantial psychological demands, which, if unaddressed, may predispose athletes to maladaptive outcomes. These dual facets underscore the necessity of conceptualizing tennis participation as both a preventive mental health-promoting activity and a context requiring carefully calibrated psychological support.

Although formal quality appraisal was not conducted, notable methodological heterogeneity was observed among the included studies. Sample sizes ranged from small pilot trials to large-scale observational. Research designs varied from randomized controlled interventions and quasi-experimental studies to qualitative case analyses. Psychological outcomes were assessed using diverse instruments, including the Beck Depression Inventory (BDI), State-Trait Anxiety Inventory (STAI), Mental Toughness Questionnaire (MTQ), and qualitative interviews, introducing variability in measurement sensitivity and comparability. Some studies lacked control groups or blinding, potentially introducing performance and selection biases. These differences in methodological rigor and design highlight the need for cautious interpretation and suggest that future research would benefit from greater standardization in measurement tools and reporting practices.

The mental health benefits of tennis participation are well-supported by the extant literature; however, the research is still scarce. Structured tennis engagement has been shown to reduce symptoms of depression and anxiety, improve self-confidence, and foster prosocial behaviors [17,21,22,41]. The reductions in psychological distress reported by Yazici et al. are particularly notable for their breadth, affecting multiple domains of psychological functioning and demonstrating that tennis may exert systemic mental health benefits beyond mood regulation alone [21]. These effects appear to be partially mediated by the social structure of tennis participation, as demonstrated by Sun et al., whose findings point to perceived social support as a key intermediary linking sustained participation with psychological resilience and prosociality [22]. This mediating role aligns with well-established theoretical frameworks such as the stress-buffering hypothesis, suggesting that social connectedness derived from sport participation enhances coping efficacy and mitigates the psychological impact of stress [42].

At the population level, survey data corroborate these individual-level findings by illustrating that tennis players report higher levels of optimism, stress tolerance, and cognitive focus relative to non-participants [17,41,43]. This suggests that the psychological benefits of tennis are both scalable and generalizable, positioning the sport as a potential public health tool for promoting psychological resilience.

Despite these benefits, participation in competitive tennis introduces distinct and measurable psychological challenges [23,24,26,35,36,39]. Competitive tennis inherently involves repeated exposure to high-pressure scenarios and performance-based evaluation. Cowden et al. highlighted the moderating role of mental toughness and resilience in buffering these stressors [23]. The inverse relationship between mental toughness and stress, coupled with the predictive role of resilience subscales, suggests that these psychological constructs function as adaptive resources enabling sustained performance in high-stakes environments. This capacity for stress modulation is particularly critical given the cumulative nature of performance pressure, as demonstrated by Harris et al., where prior errors exacerbate attentional lapses and elevate the risk of subsequent mistakes [26]. Such error cascades underline the necessity of psychological interventions targeting attentional control and emotional regulation to preserve cognitive efficiency during heightened stress.

Psychological vulnerabilities within professional tennis have also been observed, with Marazziti et al. reporting elevated obsessive–compulsive tendencies and behavioral addictions in professional players [25,38]. These tendencies likely arise from the rigid routine adherence, perfectionistic standards, and hypervigilance required for elite performance. While such traits may enhance short-term precision and consistency, they risk evolving into maladaptive patterns without appropriate psychological oversight. This highlights a key tension between the performance-enhancing and potentially detrimental aspects of cognitive rigidity in professional sports.

In developmental and youth contexts, psychological profiles appear to vary by gender [27,28,31,33,36,37]. Mourtzios et al. identified higher concentration and focus in boys and stronger emotional control and motivation in girls, suggesting that mental skills training should be tailored to align with gender-specific psychological strengths and vulnerabilities [33]. These gender-specific findings are consistent with broader developmental psychology literature emphasizing differential cognitive and emotional development trajectories between male and female athletes, and hold important implications for applied practice in youth athlete development. Psychological training programs may benefit from adopting gender-informed approaches, tailoring interventions to align with the emotional regulation strengths often observed in female athletes and the attentional and concentration-related strengths commonly seen in male athletes. For example, programs targeting performance anxiety or emotional control could emphasize reflective and cognitive-emotional strategies for girls, while interventions aimed at enhancing focus and managing distraction may be prioritized for boys. Integrating such differentiated strategies into coaching curricula, mental skills workshops, and individualized athlete monitoring systems may enhance the effectiveness of psychological support and foster more equitable and responsive development environments.

Psychological interventions have demonstrated considerable potential in supporting mental health and performance outcomes. The efficacy of mindfulness-based protocols, cognitive-behavioral skills training, and motivational self-talk underscores the malleability of psychological factors underpinning performance and well-being [28,29,30,31,32,41]. Importantly, Taylor et al. demonstrated that the systematic use of psychological preparation techniques is not only common among Olympians but is also associated with medal success, reinforcing the necessity of integrating psychological training into athletic development pathways [34]. The consistent finding that medalists are more proficient in applying psychological strategies further suggests that psychological skills are not merely ancillary to physical preparation but are fundamental determinants of elite performance outcomes.

Nonetheless, the current literature is constrained by methodological limitations that inhibit a comprehensive understanding of the long-term and causal effects of tennis on mental health. The dominance of cross-sectional designs limits temporal inference, and the scarcity of longitudinal studies precludes assessments of sustained psychological adaptation or deterioration across athletic careers. Furthermore, research disproportionately focuses on competitive and elite populations, with recreational participants underrepresented despite their substantial demographic prominence. The absence of validated, tennis-specific mental health assessment instruments remains a methodological gap, with current studies often relying on general psychological scales that may lack sensitivity to the unique stressors, performance expectations, and interpersonal dynamics inherent in tennis. Additionally, the overrepresentation of male participants in numerous studies limits the capacity to detect gender-specific patterns of psychological response and adaptation. Given the evidence of gender-related differences in psychological profiles and coping strategies, future research must prioritize balanced gender representation to enhance external validity. Moreover, although psychological interventions have shown promise, the lack of large-scale randomized controlled trials evaluating long-term effectiveness, optimal delivery modalities, and scalability represents a significant area for future inquiry.

Tennis participation emerges as a dual-context environment that can substantially enhance psychological well-being while also imposing unique psychological challenges, particularly at competitive and elite levels. Future research should focus on developing longitudinal designs, integrating recreational populations, addressing gender imbalances, and creating validated tennis-specific psychological assessment tools. Additionally, systematic evaluations of intervention efficacy and translational efforts to integrate psychological training into coaching curricula will be essential in optimizing both the mental health and performance trajectories of tennis players across all participation levels.

### The Resilience Racket Model

Drawing from the evidence synthesized in this review, we propose the *Resilience Racket Model* as an innovative conceptual framework designed to address the multifaceted demands of tennis and the systematic development of psychological resilience. This model employs a metaphorical structure wherein the components of a tennis racket represent the interconnected physical, cognitive, emotional, and environmental factors essential for optimal performance and psychological well-being in tennis athletes.

- The **handle** of the racket represents the foundational proficiencies and physical conditioning required for sustained performance. This component encompasses the athlete’s physical competencies, including cardiovascular endurance, muscular strength, agility, flexibility, and neuromuscular coordination. These foundational attributes form the base upon which psychological skills and resilience capacities are constructed. Without adequate physical preparedness, the efficacy of psychological resilience strategies may be compromised, highlighting the necessity of a robust physical foundation as the cornerstone of the model.
- The **strings** symbolize the core psychological resilience elements interwoven into the athlete’s mental framework. Each string represents a distinct resilience competency, including stress management skills, cognitive adaptability, emotional regulation, attentional control, and recovery processes. Techniques such as mindfulness, visualization, cognitive reframing, and goal-setting constitute critical elements of this dimension. The tension and balance among these psychological competencies determine the athlete’s ability to respond adaptively to the pressures inherent in both training and competitive environments.
- The **frame** of the racket represents the broader supportive infrastructure that surrounds the athlete. This includes coaching methodologies that foster psychological safety, organizational policies promoting mental health, family and peer support systems, and access to professional psychological services. Furthermore, community integration through club affiliations and virtual platforms provides avenues for social connection and mitigates the isolation often experienced in individual sports. The frame underscores that psychological resilience is not solely an individual attribute but is significantly influenced by systemic and environmental factors.
- At the center lies the **sweet spot**, conceptualized as the equilibrium nexus where physical readiness, psychological resilience, and environmental support converge. The sweet spot represents the athlete’s capacity to achieve optimal performance while maintaining psychological well-being. It is within this zone that athletes demonstrate the highest levels of mental fortitude, adaptability, and sustainable performance.

The *Resilience Racket Model* advances current theoretical paradigms by integrating physical conditioning, psychological skill development, and systemic support structures into a unified, dynamic schema. It advocates for a holistic approach to athlete development, wherein mental health is afforded equal priority alongside physical training and technical refinement. The model serves as a practical blueprint for coaches, sport psychologists, and athletes, offering a structured framework to guide the cultivation of resilience throughout athletic careers. Ultimately, the *Resilience Racket Model* aims to foster individuals who are not only physically proficient but also psychologically robust, equipping them to thrive both within the sporting context and in broader life domains.

## 5. Conclusions

This review highlights the substantial and multidimensional relationship between tennis and mental health across diverse populations and competitive levels. The evidence demonstrates that tennis engagement is associated with significant psychological benefits, including reductions in depressive and anxiety symptoms, improvements in self-confidence, and the development of resilience and prosocial behaviors. These effects are mediated not only by individual psychological competencies but also by the social and environmental structures in which athletes are embedded. The findings emphasize that tennis serves not only as a vehicle for physical fitness but also as a platform for fostering mental well-being, particularly when participation occurs in socially supportive environments.

At the same time, the review underscores the considerable psychological demands inherent in competitive and elite tennis. Elevated stress levels, vulnerability to obsessive–compulsive tendencies, and performance decrements under pressure illustrate that psychological resilience is a critical determinant of sustained success in tennis. The capacity to manage situational stress, recover from setbacks, and maintain attentional control under cumulative pressure conditions is essential for optimal performance and long-term psychological health.

While psychological interventions such as mindfulness training, cognitive-behavioral skills development, and motivational self-talk have demonstrated efficacy in enhancing mental health and performance outcomes, the current literature remains constrained by methodological limitations. These include a predominance of cross-sectional designs, underrepresentation of recreational populations, gender imbalances, and the absence of validated tennis-specific psychological assessment tools. Future research must prioritize longitudinal study designs, broader population inclusivity, balanced gender representation, and the systematic evaluation of psychological interventions tailored to the unique demands of tennis.

In response to these complexities, this review introduces the *Resilience Racket Model* as a novel conceptual framework. This model synthesizes the physical, psychological, and environmental determinants of resilience in tennis, offering a structured model for athlete development. By metaphorically aligning the elements of a tennis racket with foundational physical capacities (handle), psychological resilience competencies (strings), systemic support structures (frame), and the optimal performance nexus (sweet spot), the *Resilience Racket Model* advocates for an integrated, holistic approach to mental health in tennis. Future translational research should explore how this model can be operationalized in practice, such as embedding its components into athlete monitoring platforms, coach training programs, and youth development curriculums.

In conclusion, tennis represents a powerful context for the promotion of psychological well-being, yet its inherent demands necessitate deliberate and structured psychological support. Integrating evidence-based psychological training, combined with attention to systemic and environmental factors, is essential for fostering resilience and sustaining performance. The *Resilience Racket Model* provides a comprehensive framework for coaches, athletes, and mental health professionals to operationalize this integration, ensuring that psychological health is an essential pillar of athlete development alongside physical preparation and technical skill. 

## Figures and Tables

**Table 1:** Inclusion and Exclusion Criteria of included studies.

Eligibility Criteria	Details
Inclusion Criteria	- Peer-reviewed articles published between January 2000 and February 2025 - Articles written in English, French, or Greek - Studies focusing on tennis players (recreational to elite) - Research examining mental health outcomes, psychological factors, or interventions - Study types: empirical studies, systematic reviews, scoping reviews, or meta-analyses
Exclusion Criteria	- Studies not specific to tennis (e.g., mixed sports data without separate tennis analysis) - Non-peer-reviewed literature (e.g., theses, opinion pieces, conference abstracts) - Articles focusing solely on physical performance without any psychological assessment - Articles unavailable in full text

## Data Availability

No new data were created.

## Abbreviations

The following abbreviation is used in this manuscript:

PRISMA-ScR	Preferred Reporting Items for Systematic reviews and Meta-Analyses extension for Scoping Reviews
